# Evaluation of Solutions Containing Fluoride, Sodium Trimetaphosphate, Xylitol, and Erythritol, Alone or in Different Associations, on Dual-Species Biofilms

**DOI:** 10.3390/ijms241612910

**Published:** 2023-08-18

**Authors:** Igor Zen, Alberto Carlos Botazzo Delbem, Tamires Passadori Martins, Leonardo Antônio de Morais, Caio Sampaio, Thayse Yumi Hosida, Douglas Roberto Monteiro, Juliano Pelim Pessan

**Affiliations:** 1 Department of Preventive and Restorative Dentistry, School of Dentistry, Araçatuba, São Paulo State University (UNESP), Rua José Bonifácio, 1193, Araçatuba 16015-050, SP, Brazil; igorzen@gmail.com (I.Z.); alberto.delbem@unesp.br (A.C.B.D.); tamires.passadori@unesp.br (T.P.M.); leonardo.a.morais@unesp.br (L.A.d.M.); caio.sampaio@unesp.br (C.S.); thayse.hosida@unesp.br (T.Y.H.); douglas@unoeste.br (D.R.M.); 2 Postgraduate Program in Health Sciences, University of Western Saão Paulo (UNOESTE), Presidente Prudente 19050-920, SP, Brazil

**Keywords:** biofilms, *Streptococcus mutans*, *Candida albicans*, phosphates, sugar alcohols, fluorides, xylitol, erythritol, dental caries

## Abstract

Although the association of polyols/polyphosphates/fluoride has been demonstrated to promote remarkable effects on dental enamel, little is known on their combined effects on biofilms. This study assessed the effects of solutions containing fluoride/sodium trimetaphosphate (TMP)/xylitol/erythritol on dual-species biofilms of *Streptococcus mutans* and *Candida albicans*. Biofilms were grown in the continuous presence of these actives alone or in different associations. Quantification of viable plate counts, metabolic activity, biofilm biomass, and extracellular matrix components were evaluated. Overall, fluoride and TMP were the main actives that significantly influenced most of the variables analyzed, with a synergistic effect between them for *S. mutans* CFUs, biofilm biomass, and protein content of the extracellular matrix (*p* < 0.05). A similar trend was observed for biofilm metabolic activity and carbohydrate concentrations of the extracellular matrix, although without statistical significance. Regarding the polyols, despite their modest effects on most of the parameters analyzed when administered alone, their co-administration with fluoride and TMP led to a greater reduction in *S. mutans* CFUs and biofilm biomass compared with fluoride alone at the same concentration. It can be concluded that fluoride and TMP act synergistically on important biofilm parameters, and their co-administration with xylitol/erythritol significantly impacts *S. mutans* CFUs and biomass reduction.

## 1. Introduction

Dental caries is a biofilm-mediated and diet-modulated disease, which results in the loss of the mineral dental tissues. Caries lesions result from a dynamic process, whose main causative factor are poor oral hygiene, bad eating habits (i.e., high sugar consumption), and an alteration of oral bacterial flora [1,2]. One commensal member of all microorganisms related to the onset of this disease is *Streptococcus mutans*, which is found in caries lesions of both adults and children [3]. Furthermore, *Candida albicans* is a commensal fungus found in 96% of children with dental caries [4], and oral cavities already colonized by this fungus may have nearly five times a greater risk of developing early childhood caries [5]. Furthermore, *S. mutans* can produce anchored proteins that allow and facilitate their binding to *C. albicans*, leading to a more virulent environment in the biofilm [6].

The use of fluoride (F) products plays an important role in controlling and arresting dental caries. Among the most used formulations, there is strong evidence on the clinical efficacy of toothpastes containing ~1100 ppm F or above in controlling caries [7,8], with a lower number of trials attesting the efficacy of formulations with lower F content. Furthermore, data have shown that the addition of sodium trimetaphosphate (TMP) to low-F toothpastes promotes synergistic effects on dental hard tissues [9]. Moreover, TMP presents effects over dual-species biofilms of *S. mutans* and *C. albicans*, acting directly on the metabolic activity and production of biofilm biomass, and reducing the protein, carbohydrate, and DNA content of the extracellular matrix [10].

In addition to the actives above, natural products such as xylitol and erythritol have demonstrated marked effects on biofilms [11,12]. Both polyols have been shown to decrease biofilm acidogenicity and volume and to inhibit the growth of *S. mutans* [13]. The effects of these polyols over the extracellular matrix have also been shown to decrease the microorganism’s adherence to the enamel surface and to decrease the expression of genes involved in the metabolism of sucrose [14].

Given the different mechanisms of action of F, TMP, xylitol, and erythritol on cariogenic-related biofilms, it would be possible that the association of these actives could increase the antibiofilm effects of topically applied formulations. Thus, this study aimed to assess the effects of F, TMP, xylitol, or erythritol, alone or in different combinations, on dual-species biofilms of *S. mutans* and *C. albicans*, using different biofilm quantification assays (cultivable cells, total biomass, metabolic activity, and extracellular matrix composition). The study’s null hypothesis was that the effects of the actives alone would not be significantly different from those observed by different associations on the variables analyzed.

## 2. Results

**Figure 1:**
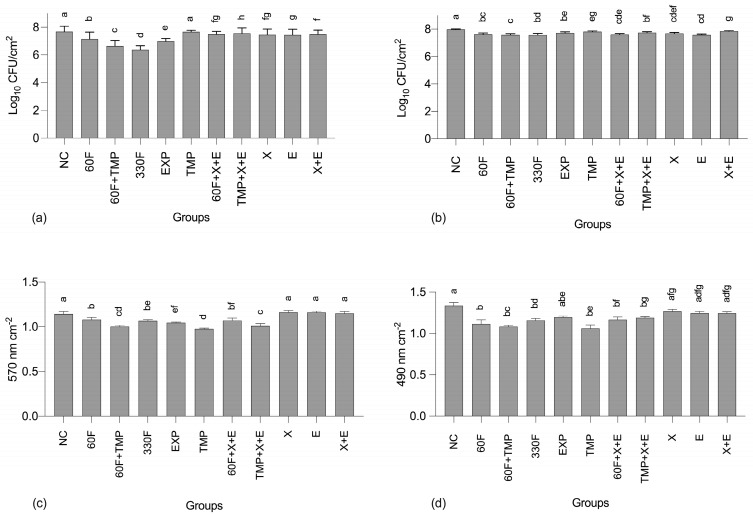
Logarithm of colony-forming units cm^2^ for *S. mutans* (**a**) and *C. albicans* (**b**), and absorbance values cm^2^ obtained for the total biomass (**c**) and metabolic activity (**d**) quantification assays. Error bars denote the SD of the means. Different lowercase letters symbolize statistical differences among the groups (*p* < 0.05, *n* = 9). NC: negative control (untreated biofilms), “60F” = 60 ppm F; “330F” = 330 ppm F; “X” = 4.8% Xylitol; “E” = 1.2% Erythritol.

Regarding the number of viable cells of *S. mutans*, all test groups had significantly lower values compared to the NC, except for TMP alone (Figure 1a). Overall, the largest reductions were observed for 330F, followed by 60F+TMP, EXP, and 60F, with a synergistic effect observed between TMP and 60F. For *C. albicans*, all test groups had significantly lower numbers of viable cells compared to the NC, with no clearly defined trend among groups containing the actives (Figure 1b).

Solutions containing TMP, TMP+X+E, or 60F+TMP promoted significantly larger reductions in the total biofilm mass compared to the other groups (Figure 1c). On the other hand, solutions containing only the polyols (xylitol or erythritol), combined or alone, were not capable of promoting significant reductions compared to the NC. A similar trend was observed for the metabolic activity of the biofilms assessed (Figure 1d).

All test solutions were able to reduce the protein and carbohydrate contents of the extracellular matrix of the biofilms compared to the NC (Table 1), with the largest effects seen for 330F and 60F, in a dose–response pattern, followed by TMP and 60F+TMP. As for DNA content, the EXP was the only polyol-containing solution capable of reducing this component; all other solutions containing the polyols led to DNA values similar to the NC. For all other groups containing TMP and/or F, significantly lower DNA values were observed in comparison to NC.

## 3. Discussion

The high consumption of fermentable sugars in a diet, in combination with insufficient dental hygiene, creates favorable conditions for the growth and development of cariogenic biofilms, especially at stagnation sites [1]. To overcome this issue, promising strategies based on F-based formulations have been tested in association with new preventive/therapeutic agents, which have been shown to promote significant effects on enamel de- and re-mineralization, as well as in the reduction of biofilm formation and, consequently, dental caries [15]. Within this context, the present study evaluated the effects of solutions containing F, TMP, X, and E, alone or in different combinations, demonstrating that most of the associations affected several variables related to dual-species cariogenic-related biofilms of *S. mutans* and *C. albicans*, thus leading to partial rejection of the null hypothesis.

Among the more than 700 different microbial species that inhabit the oral cavity, *S. mutans* is one of the main cariogenic pathogens, which is related to the onset of caries lesions [16,17,18]. The present findings showed that the major effect on *S. mutans* CFU counts was promoted by F, in a dose–response manner (Figure 1a). Such effects were somehow expected considering the action of F on this bacterium by inhibiting the activity of the glycolytic enzymes [19], which might be potentiated by the constant presence of this active during initial biofilm formation and growth. Interestingly, such effects seem to have affected all the other variables analyzed, to a lesser extent and not following a dose–response trend though. As for TMP, it was noteworthy that despite that this polyphosphate promoted no reduction in *S. mutans* CFU counts when administered alone (compared with the negative control), it potentiated the effects of F. In fact, similar results were found by Cavazana et al. [10] using the same biofilm model but administering the actives at higher concentrations and in short-term exposure times (instead of its constant presence during biofilm formation).

Synergism between the actives has been extensively reported for enamel de- and re-mineralization and ion dynamics administered in different vehicles [9,20,21]. These studies suggest that TMP’s mechanism of action is related to its ability to adsorb onto the enamel surface, which hinders acid diffusion and decreases enamel demineralization. In the present study, however, the absence of tooth enamel in the model suggests that the positive effects of F in combination with TMP on the biofilms assessed might mostly be attributed to the inhibition of acidic niche formation and cell adhesion and aggregation due to the action of TMP [22]. Once again, this hypothesis is supported by a previous study using the same dual-species model as in the present work, showing an enhanced buffering effect of F combined with TMP in comparison to the actives administered alone [20].

The aforementioned synergism of F + TMP on CFU counts of *S. mutans* was, in general, reflected in all the other parameters assessed, including biofilm biomass, metabolic activity, and, to a lesser extent, the protein content of the extracellular matrix. Interestingly, however, the highest effects on biofilm biomass and metabolic activity were not promoted by F, but instead by TMP, and this polyphosphate was also shown to potentiate the effects of the polyols on both variables analyzed (Figure 1c,d). Regarding the extracellular matrix, TMP and F (administered alone or in combination) also promoted significant reductions in protein, carbohydrate, and DNA contents. Following a similar rationale to that described for F, it seems plausible that the effects of TMP on biofilm biomass and metabolic activity resulted in a lower content of the extracellular matrix components, with a synergistic effect with F on its protein content (Table 1). These findings are in line with previous data obtained by the use of F + TMP as treatment solutions, which demonstrated significant reductions in total biomass values, metabolic activity, and extracellular matrix components in mixed biofilms of *S. mutans* and *C. albicans* [10]. In addition, the administration of toothpastes containing these actives on polymicrobial biofilms formed in situ resulted in a lower production of extracellular polysaccharides compared with F alone [9]. A possible explanation for these reductions is related to a slight chelating ability of TMP [23], which allows its binding to the cell wall of gram-positive bacteria, specifically through Ca^+^ and Mg^+^ ions, thus leading to changes in bacterial metabolism [24,25]. Furthermore, this active may affect the expression of genes associated with phosphate and amino acid biosynthesis in microbial cells [26]. This reduction may also be associated with the bioavailability of nutrients in the culture medium, since possible interactions between TMP and ions present in the medium may occur, causing a reduction in biofilm metabolism [10,27].

As for xylitol and erythritol, the data analyzed together point out very modest effects of these polyols on the biofilms compared with the negative control. Although at first glance the trend observed was not expected, the sucrose content in the culture medium and the maturation stage of the biofilms likely played major roles in the results obtained. Given that sucrose was not administered as pulses (to mimic cariogenic challenges), but instead, it was constantly present in the culture medium, bacteria would predominantly metabolize fructose and glucose (from sucrose) over sugar-alcohols, so it seems unlikely that the polyols would be metabolized in the presence of hexoses [28,29]. Similar results have been previously reported for single- and dual-species biofilms of *S. mutans* and *C. albicans* at mature formation stages [30,31]. Conversely, biofilms at early formation stages (8–24 h) were shown to be more susceptible to treatments with xylitol and erythritol compared with mature biofilms [12,32]. Therefore, the time for biofilm formation in the present study (96 h) might also have influenced the results obtained concerning the effects of xylitol and erythritol.

Despite the small effects of the polyols on CFU counts, biofilm biomass, and on protein and DNA contents of the extracellular matrix, these actives promoted reductions of ~30% in the carbohydrate concentrations of the biofilms (Table 1). This is important from a clinical perspective, as this parameter is directly related to the biofilm’s virulence [33]. Data on the metabolic activity also deserve comment, since the results from biofilms treated with the polyols were as high as those observed for the negative control. Analyzed together, these two parameters are in line with the well-known futile cycle of the polyols [34], given that the presence of the sugar alcohols led to highly active biofilms (also resulting in high biofilm biomass) but a significantly lower reserve of extracellular carbohydrates in the biofilm matrix.

The negligible effect of the actives on *C. albicans* cells (Figure 1b) must also be addressed. Treatment with F and/or TMP has already been shown to be ineffective on this fungus [10,20], and the effects of polyols have usually been described for bacteria, so the trend observed was not necessarily unexpected. It should be noted that *C. albicans* was used in the present study because this dual-species biofilm model (i.e., *S. mutans* and *C. albicans*) was previously validated as a cariogenic model of relatively low complexity, able to reproduce the pH changes following sucrose exposure similarly to what is observed in vivo [20]. This model has successfully been used to assess the effects of F, alone or associated with polyphosphates, as well as antifungal drugs [10,20]. However, this model has not been previously used to assess the effects of polyols, so some aspects deserve comment, especially the complexity of this fungus and the different size of the microorganism structure compared to prokaryotic cells [35]. Moreover, when sucrose is present in the medium, a higher number of hyphae cover the biofilm, which leads to a denser structure that hinders the access of actives to act over the biofilm cells [28,36]. Furthermore, *S. mutans* seems to stimulate the growth and consequently increase the amount of *C. albicans* cells in the biofilm, making it more difficult for the active compounds to affect the biofilm cells. Considering that polymicrobial biofilms formed in vivo may present a less dense structure than that observed for the dual-species model used in the present study, our results must be interpreted in light of these considerations.

Finally, despite the apparent low effect of the polyols on some of the variables analyzed, it must be emphasized that the reductions in the CFU counts, biofilm biomass, and proteins and carbohydrates were significant when compared with the negative control. Most importantly, the association of the polyols with the other two actives (i.e., the experimental group) resulted in similar effects to those observed for the positive control (330F) regarding biofilm biomass and some of the extracellular matrix components. This is paramount from a clinical standpoint, especially considering the effects of the polyols on enamel de- and re-mineralization [37,38,39], given their ability to form complexes with calcium ions and induce remineralization of the deeper layers of enamel lesions [40,41]. Thus, despite the limitations of in vitro biofilm models, the present results could have important implications on caries dynamics, as the simultaneous administration of the four actives could be effective on enamel de-/re-mineralization (shown in previous studies) and on biofilm’s virulence (as in the present study). Further studies using more complex biofilm models, especially formed on mineralized dental substrates, could provide additional data on the actual effects of the actives alone and/or associated, considering both the biofilm and mineralized tissues together. In addition, as future steps, it could be interesting to investigate the combined effects of the actives studied in this work with novel promising technologies such as biomimetic hydroxyapatite, which are the resultants of strategies aiming to shift from enamel remineralization to regeneration, as successfully described in previous studies [42,43].

## 4. Materials and Methods

### 4.1. Preparation of the Test Solutions

The solutions were prepared by weighing and diluting the following compounds: xylitol (Sigma-Aldrich, St. Louis, MO, USA), erythritol (Sigma-Aldrich), trisodium trimetaphosphate (Sigma-Aldrich), or sodium fluoride (NaF, Sigma-Aldrich), alone or in different associations, in order to achieve final concentrations of 4.8%, 0.6%, 0.075%, and 60 ppm F, respectively. The experimental groups in this study were: Pure AS (negative control = “NC”), NaF 330 ppm F (positive control–“330F”), Xylitol 4.8% (“X”), Erythritol 1.2% (“E”), Sodium trimetaphosphate 0.075% (“TMP”), NaF 60 ppm F (“60F”), “X+E”, “60F+TMP”, “TMP+X+E”, “60F+X+E”, and experimental solution containing 60F+TMP+X+E (“EXP”). All compounds were filter-sterilized to obtain an aseptic condition. The concentrations were adopted to achieve 30% of the initial concentrations used in a previous study, administered as dentifrices [37].

### 4.2. Growth Conditions

The biofilms were formed in sucrose-containing artificial saliva (AS) as a culture medium, according to the following composition for 1.0 l deionized water: 4 g of sucrose, 2 g of yeast extract, 5 g of bacteriological peptone, 1 g of mucin type III, 0.35 g of NaCl, 0.2 g of CaCl_2_, and 0.2 g of KCl. The pH of the solution was adjusted with NaOH to 6.8 [20,44]. All reagents for the preparation of AS were purchased by Sigma-Aldrich.

Strains from the American Type Culture Collection (ATCC) were adopted in this work: *C. albicans* ATCC 10231 and *S. mutans* ATCC 25175. For *C. albicans*, colonies cultured on Sabouraud dextrose agar (SDA; Difco, Le Pont de Claix, France) were suspended in 10 mL of Sabouraud dextrose broth (Difco) and incubated overnight in aerobioses at 120 rpm (37 °C). *S. mutans* colonies grown on brain heart infusion agar (BHI Agar; Difco) were suspended in 10 mL of BHI broth (Difco) and statically incubated overnight in 5% CO_2_ at 37 °C. Afterward, the cells were centrifuged (8000 rpm, 5 min), and the pellets were washed twice with 10 mL of 0.85% NaCl. The number of *C. albicans* cells was adjusted to 1 × 10^7^ cells/mL in AS using a Neubauer counting chamber, and the number of *S. mutans* cells was spectrophotometrically (640 nm) adjusted to 1 × 10^8^ cells/mL [20].

### 4.3. Biofilm Formation and Growth in the Presence of the Test Solutions

The *S. mutans* and *C. albicans* dual biofilms were grown in flat-bottom 96- or 6-well microtiter plates (Costar, Tewksbury, MA, USA) for the quantification assays or the analysis of the components of the extracellular matrix, respectively.

For the quantification assays, 100 µL of each microbial suspension (2 × 10^7^ cells mL for *C. albicans*, 2 × 10^8^ cells mL for *S. mutans*) was added to the wells, and the plates were incubated in 5% CO_2_ at 37 °C for 2 h. Next, AS was removed, and the wells were washed once with 0.85% NaCl. Each test solution was added to AS to achieve a final solution of 100 µL. These compounds were then pipetted in the walls containing adhered cells, and the plates were incubated for 24 h in 5% CO_2_ at 37 °C in order to allow biofilm formation [45]. The medium was renewed every 24 h by removing 100 mL and adding an equal volume of a fresh test solution. After completing the 96 h biofilm formation, the test solutions were removed from the wells, and the resulting biofilms were washed once with 0.85% NaCl to eliminate planktonic cells.

Regarding the analysis of the components of the extracellular matrix, 2 mL of each microbial suspension (2 × 10^7^ cells mL for *C. albicans*, 2 × 10^8^ cells mL for *S. mutans*) was added to the wells, and the plates were incubated in 5% CO_2_ at 37 °C for 2 h. Next, AS was removed, and the wells were washed once with 0.85% NaCl. Each test solution was added to AS to achieve a final solution of 4 mL. The compounds were added as described above, and the medium was renewed every 24 h by removing 2 mL and adding an equal volume of fresh test solution. After completing the 96 h biofilm formation, the test solutions were removed from the wells, and the resulting biofilms were washed once with 0.85% NaCl to eliminate planktonic cells.

### 4.4. Quantification Assays

#### 4.4.1. Cultivable Cells

The quantification of cultivable cells was evaluated by colony-forming units counts (CFUs), as previously described [10]. Briefly, the resulting biofilms after the last treatment were resuspended in 0.85% NaCl and scraped from the wells. Biofilm suspensions were then serially diluted in 0.85% NaCl and plated on CHROMagar *Candida* (Difco), and BHI agar was supplemented with 7 µL mL amphotericin B (Sigma-Aldrich) for *C. albicans* and *S. mutans* counts, respectively. The plates were incubated for 48 h at 37 °C. The CFUs were expressed as log_10_CFU/cm^2^.

#### 4.4.2. Total Biofilm Biomass

Biofilm biomass was assessed by the crystal violet (CV) staining assay [46]. In brief, the biofilms were fixed for 15 min at room temperature with 99% methanol (Sigma-Aldrich), stained for 5 min with 1% CV (Sigma-Aldrich), and de-stained through exposure to 33% acetic acid (Sigma-Aldrich). Absorbance values were read at 570 nm and represented as a function of the area of the wells (absorbance cm^2^). Wells containing AS without microbial cells were used as blanks.

#### 4.4.3. Metabolic Activity

The evaluation of the metabolic activity of biofilm cells was performed by the 2,3-bis (2-methoxy-4-nitro-5-sulfophenyl)-5-[(phenylamino)carbonyl]-2H-tetrazolium hydroxide (XTT; Sigma-Aldrich) reduction method. In summary, XTT and phenazine methosulphate (Sigma-Aldrich) solutions were pipetted into the wells after being combined. The microtiter plates were then incubated (37 °C, 120 rpm) for 3 h (protected from light), and the absorbance values were measured at 490 nm (absorbance cm^2^). Blanks were processed as described for the total biomass assay [10].

### 4.5. Analysis of Extracellular Matrix Composition

For this assay, dual-species biofilms were grown in the six-well plates (Costar) containing 4 mL of the microbial suspension, as described above. After 96 h with the solutions, the biofilms were resuspended in 0.85% NaCl, scraped from the wells, and the liquid phase of the extracellular matrix was extracted by sonication (for 30 s at 30 W), as detailed elsewhere [47]. The bicinchoninic acid method (Kit BCA; Sigma-Aldrich) was performed for protein determination of the extracellular matrix, using bovine serum albumin as the standard [47], while the carbohydrate content was measured using the method devised Dubois et al. [48], with glucose as the standard. For DNA content, a volume of 1.5 mL of the liquid phase of the extracellular matrix was spectrophotometrically analyzed (at 260 and 280 nm) in a Nanodrop Spectrophotometer (EONC Spectrophotometer of EONC, Biotek, Winooski, VT, USA) [27]. Protein, carbohydrate, and DNA values were expressed as mg g dry weight of biofilm.

### 4.6. Statistical Analysis

All experiments were conducted in biological triplicate on three different days. The normality of the data was verified by Shapiro–Wilk’s test. All data passed normality and were submitted to one-way ANOVA and Tukey’s HSD post hoc test. All analyses were performed with a significance level of 5% using SigmaPlot 12.0 software (Systat Software Inc., San Jose, CA, USA).

## 5. Conclusions

Based on the results obtained, it is possible to conclude that F and TMP present significant antibiofilm effects and that these actives have a synergistic effect on some of the parameters analyzed. The administration of the polyols, however, promoted an overall modest effect, but their co-administration with fluoride and TMP was able to promote significant reductions in biofilm biomass. The results presented in this study provide important insight into how the combination of phosphates, polyols, and F administered as solutions act on cariogenic-related biofilms of *S. mutans* and *C. albicans* in vitro.

## Figures and Tables

**Table 1:** Mean (standard deviation of the means) proteins, carbohydrates, and DNA (mg/g of biofilm dry weight) in the extracellular matrix of dual-species biofilms of *S. mutans* and *C. albicans* obtained after treatment with different concentrations of fluoride, sodium trimetaphosphate, xylitol, or erythritol, alone or in different associations.

Matrix Component	Groups										
	NC	60F	60F+TMP	330F	EXP	TMP	60F+X+E	TMP+X+E	X	E	X+E
Proteins	31.37 ^A^ (1.97)	24.75 ^B^ (1.14)	22.57 ^C^ (1.76)	20.71 ^D^ (1.77)	23.62 ^BC^ (1.31)	22.15 ^D^ (1.80)	24.81 ^B^ (1.71)	27.18 ^E^ (1.60)	28.08 ^E^ (1.81)	27.95 ^E^ (1.00)	28.57 ^E^ (1.20)
Carbohydrates	556.4 ^A^ (28.6)	243.0 ^BC^ (16.1)	239.0 ^CDE^ (15.9)	210.5 ^CFG^ (19.5)	246.9 ^BDG^ (25.7)	209.4 ^EF^ (14.5)	254.7 ^BD^ (23.0)	363.2 ^H^ (24.3)	405.6 ^I^ (11.7)	365.1 ^H^ (22.1)	413.2 ^I^ (23.9)
DNA	14.5 ^A^ (0.67)	12.7 ^BC^ (0.42)	13.0 ^BD^ (0.39)	9.6 ^B^ (0.55)	12.8 ^BE^ (0.43)	10.7 ^BF^ (0.34)	13.2 ^ACDEF^ (0.31)	13.6 ^ACDEF^ (0.39)	14.0 ^AD^ (0.24)	14.2 ^A^ (0.54)	14.0 ^ADE^ (0.33)

Different upper-case letters symbolize statistical differences among the groups (*p* < 0.05) (*n* = 9). NC: negative control (untreated biofilms), 60F: 60 ppm Sodium Fluoride, 60F+TMP: 60 ppm Sodium Fluoride + 0.075% Sodium trimetaphosphate, 330F: 330 ppm Sodium Fluoride, EXP: 4.8% Xylitol + 1.2% Erythritol + 60 ppm Sodium Fluoride + 0.075% Sodium trimetaphosphate, TMP: 0.075% Sodium trimetaphosphate, 60F+X+E: 60 ppm Sodium Fluoride + 4.8% Xylitol + 1.2% Erythritol, TMP+X+E: 0.075% Sodium trimetaphosphate + 4.8% Xylitol + 1.2% Erythritol, X: 4.8% Xylitol, E: 1.2% Erythritol, X+E: 4.8% Xylitol + 1.2% Erythritol.

## Data Availability

The data presented in this study are available on request from the corresponding author.
